# *Toxoplasma gondii* in beef consumed in France: regional variation in seroprevalence and parasite isolation

**DOI:** 10.1051/parasite/2019076

**Published:** 2019-12-23

**Authors:** Radu Blaga, Dominique Aubert, Anne Thébault, Catherine Perret, Régine Geers, Myriam Thomas, Annie Alliot, Vitomir Djokic, Naïma Ortis, Lénaïg Halos, Benoît Durand, Aurélien Mercier, Isabelle Villena, Pascal Boireau

**Affiliations:** 1 UMR BIPAR, Ecole Nationale Vétérinaire d’Alfort, ANSES, Université Paris-Est, INRA, National Reference Laboratory for Foodborne Parasites, Animal Health Laboratory 14 rue Pierre et Marie Curie 94700 Maisons-Alfort France; 2 National Reference Center on Toxoplasmosis, Toxoplasma Biological Resources Center, CHU Reims and EA7510, SFR CAP-Santé, University of Reims Champagne-Ardenne, USC EpiToxo ANSES 51095 Reims France; 3 ANSES, Direction de l’évaluation des risques, Unité Méthodes et Etudes 94700 Maisons-Alfort France; 4 UMR BIPAR, ANSES, Ecole Nationale Vétérinaire d’Alfort, INRA, Université Paris-Est, National Reference Laboratory for Foodborne Parasites, Animal Health Laboratory 14 rue Pierre et Marie Curie 94700 Maisons-Alfort France; 5 Epidemiology Unit, Paris-Est University, Laboratory for Animal Health, French Agency for Food, Environmental and Occupational Health and Safety (ANSES) 94700 Maisons-Alfort France; 6 INSERM, Université de Limoges, CHU Limoges, UMR 1094, Institut d’Epidémiologie et de Neurologie Tropicale, GEIST 87000 Limoges France; 7 National Reference Center on Toxoplasmosis, Toxoplasma Biological Resources Center, CHU Limoges 87042 Limoges France

**Keywords:** *Toxoplasma gondii*, Bovines, Meat, Strain isolation, Prevalences

## Abstract

In France, the consumption of cattle and sheep meat appears to be a risk factor for infection of pregnant women with *Toxoplasma gondii*. Several nation-wide surveys in France have investigated the prevalence of *T. gondii* in sheep and pig meat, but little is known at present about the prevalence of the parasite in beef. The main objective of the present cross-sectional survey was to estimate the seroprevalence of *T. gondii* infection in beef consumed in France. A secondary objective was to attempt to isolate *T. gondii* from cattle tissues and to study the geographical and age variations of this seroprevalence. The overall estimate of seroprevalence of *T. gondii* in bovine carcasses (*n* = 2912), for a threshold of 1:6 was 17.38%. A strong age effect was observed (*p* < 0.0001) with a seroprevalence of 5.34% for calves (<8 months) and 23.12% for adults (>8 months). Seroprevalence estimates given by area of birth and area of slaughtering for adults showed that the areas with the highest seroprevalence were not the same between these two variables. Only two strains, corresponding to genotype II, were isolated from heart samples, indicating that there is a limited risk of human infection with *T. gondii*, which needs to be correlated with the food habit of consuming raw or undercook (*bleu* or *saignant*) beef. However, new questions have emerged, especially concerning the isolation of parasites from beef and the precise role of bovines, generally described as poor hosts for *T. gondii*, in human infection.

## Abstract (fr)

En France, la consommation de viande bovine et ovine apparaît comme un facteur de risque pour la contamination des femmes enceintes par *Toxoplasma gondii*. Plusieurs enquêtes nationales ont été réalisées afin de déterminer le niveau de contamination par *T. gondii* de la viande ovine et porcine, en France, mais très peu est encore connu quant à la prévalence du parasite dans la viande bovine. La présente enquête transversale avait pour objectif principal d’estimer la séroprévalence de l’infection à *T. gondii* dans la viande bovine consommée en France, ainsi que d’isoler *T. gondii* à partir de tissus de bovins et d’étudier, à titre d’objectif secondaire, les variations géographiques et d’âge de cette prévalence. L’estimation globale de la séroprévalence de *T. gondii* dans les carcasses de bovins (*n* = 2912) était de 17,38 % (pour un seuil de dilution à 1:6). Un effet significatif de l’âge a été observé (*p* < 0,0001) avec une séroprévalence de 5,34 % pour les veaux (<8 mois) et de 23,12 % pour les adultes (>8 mois). Les estimations de séroprévalence données par zone de naissance et par zone d’abattage pour les adultes montrent que les zones de séroprévalence les plus élevées n’étaient pas les mêmes pour ces deux variables. Seulement deux souches, de génotype II, ont été isolées à partir d’échantillons de cœurs, soulignant que le risque d’infection humaine est limité, mais doit être corrélé avec les habitudes de consommation alimentaire de la viande bovine peu/pas cuite (bleu ou saignante). Cependant, de nouvelles questions se posent, notamment en ce qui concerne l’isolement du parasite à partir de la viande bovine, ainsi que le rôle précis des bovins, généralement décrits comme des hôtes médiocres pour *T. gondii*, dans la contamination humaine.

## Introduction

*Toxoplasma gondii* is an obligate intracellular parasite belonging to the Apicomplexa phylum, with world-wide distribution. It has three infectious stages: the tachyzoites, the bradyzoites, and sporulated oocysts (with eight sporozoites). These stages are linked in a complex life cycle, with the cat and other felids serving as the definitive hosts, while virtually all warm-blooded species, including humans, can act as intermediate hosts [53]. The main route of transmission is oral: humans and animals can become infected by ingesting either tissue cysts containing bradyzoites, from raw or undercooked meat, or sporulated oocysts, from various environmental sources (soil, water, vegetables). Consumption of undercooked infected meat may be considered a major risk factor for humans, especially in Europe, where it has been associated with 30–63% of infections [14, 56], while Hill [35] has demonstrated the predominance of oocyst-driven infections in North America. In humans, infection is usually either asymptomatic or the cause of mild flu-like symptoms. However, toxoplasmosis can be life-threatening especially in immunocompromised individuals. Moreover, if acquired during pregnancy, toxoplasmosis can cause miscarriage or congenital malformations affecting the brain, eyes or other organs of the foetus [34]. In animals, infection with *T. gondii* may cause abortion in sheep, goats and pigs [28], while in cattle, natural *T. gondii* infection does not appear to cause clinical disease or abortion [23]. Therefore, interest in *T. gondii* in cattle comes mainly from a public health perspective; if cattle carry viable tissue cysts, they may be a source of human infections. This might be particularly true in western countries, where eating raw or undercooked beef is, to some extent, related to high standards of living [28]. Moreover, several human toxoplasmosis outbreaks were recorded world-wide, with various sources of infection, four of them identifying the consumption of raw or undercooked beef as the most probable source [2, 54]. However, the quantified role of these animals in the transmission of *T. gondii* to humans remains unclear. On the one hand, serological evidence of *T. gondii* exposure in cattle has been published world-wide [2, 10, 28, 31, 36–41, 45, 47, 48, 50, 51, 55, 57], and parasite DNA has been detected by means of polymerase chain reaction (PCR) [5, 32, 49, 50]. On the other hand, a few attempts have succeeded in isolating the parasite from various types of bovine tissues, by means of bioassays [7, 23, 25, 50, 51].

In spite of a constant decrease in the last 40 years, France is still the leading consumer of beef within EU with 24 kg/inhabitant in 2013 [30]. When it comes to cooking habits, a large majority (>50%) of French adults consume undercooked (*bleu, saignant, à point*) lamb, beef or horse meat, while 20% of them eat, at least occasionally, raw beef [2]. Not surprisingly, the consumption of cattle and sheep meat has appeared as a risk factor for infection of pregnant women in France [6]. Similarly in Italy, in a recently published quantitative risk assessment model to estimate the yearly possibility of acquiring human toxoplasmosis infection due to consumption of beef or pork, beef scored higher [8]. Few previous studies [2, 31] have investigated *T. gondii* infection in cattle in France, while a nation-wide survey was conducted to evaluate the prevalence of *T. gondii* in sheep and pig meat consumed in France [19, 33].

Since several studies have pointed out the need for large-scale screening to clarify the role of cattle as carriers of viable tissue cysts [49, 50], in the present study, we used a cross-sectional survey to estimate, as a primary objective, the seroprevalence of *T. gondii* infection in beef consumed in France. As a secondary objective, we attempted the isolation of *T. gondii* from cattle tissues and studied the geographical and age variations of this seroprevalence.

## Materials and methods

### Sampling strategy

#### Sampling size

For an expected seroprevalence ranging from 5% to 43%, as previously observed in different studies in cattle [28, 31, 50, 57], a sample size of 3000 animals enabled us to guarantee a relative precision of minimum 0.2% [20]. Two different types of meat were targeted in terms of origin: national production (slaughtered in France) and imports (slaughtered within the EU or outside the EU). The relative portion of imports of fresh beef was estimated in 2007 to be 15% and 20% (Data of the French Ministry of Agriculture). The number of samples from national production was set originally at 2350 and from imports at 650.

#### Samples of French origin

**Figure 1:**
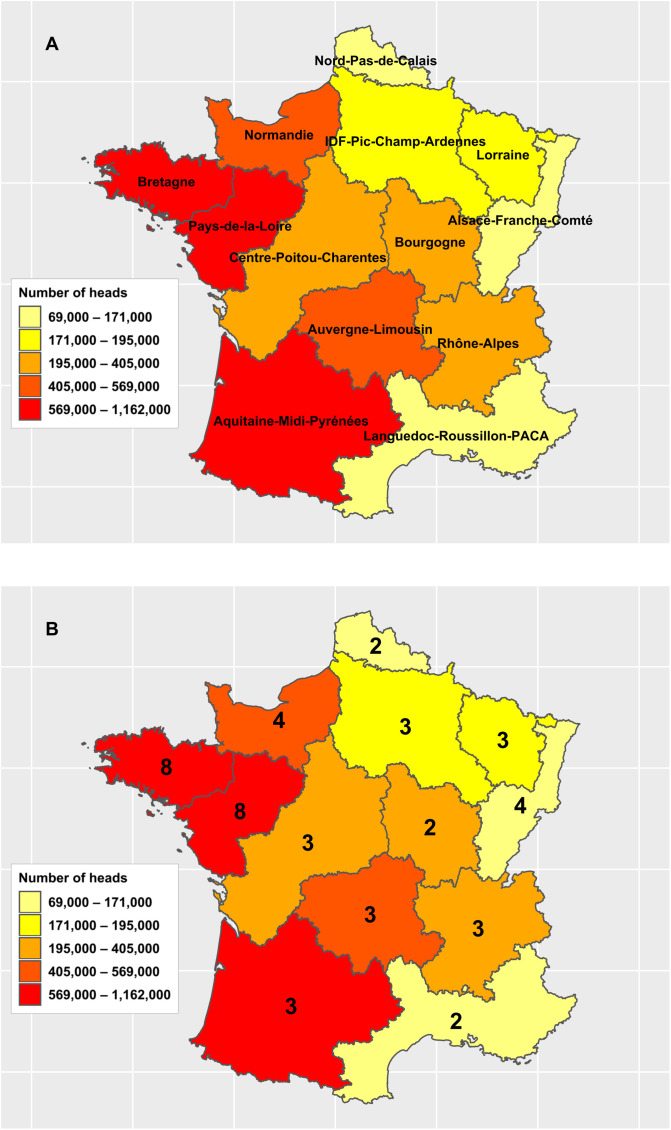
(A) Map of French beef production according to the Ministry of Agriculture database. The colour gradient represents the number of cattle slaughtered in 2007. (B) The numbers represent the number of slaughterhouses per region that were included in the cross-sectional survey of *Toxoplasma gondii* presence in beef produced in France.

The sampling strategy was established in order to be representative of the spatial variability of slaughterhouses and the age of animals. The first unit of sampling was the slaughterhouse. From French Ministry of Agriculture databases for 2007, 245 cattle slaughterhouses were identified. The second unit of sampling was 50 animals in a single week from the same slaughterhouse for logistical reasons. In order to obtain enough samples from the 245 slaughterhouses, we first excluded 111 with less than 5200 animals/year (less than 100 per week), which represented 4.66% of production. We also excluded two overseas slaughterhouses (to limit costs). The 25 largest slaughterhouses were included *a priori* in the sampling, representing 50.94% of meat production. We selected a minimum of one slaughterhouse per administrative region (*n* = 21), with probability of sampling proportional to meat production (unequal probability of sampling) using the SURVEYSELECT procedure in SAS. One slaughterhouse was added to the sampling protocol and selected among three other slaughterhouses in the Pyrénées-Atlantiques department because of the high seroprevalence rate for ovine *T. gondii* infection, as shown in our previous results [33]. Therefore 47 different slaughterhouses were selected at the beginning of the study, but due to practical reasons, 48 were included (one region performed sampling in two different slaughterhouses), covering all regions of France (Fig. 1). It was also checked that the age distribution of the sampled animals was similar to that of cattle slaughtered at the national level. The percentage of calves (<8 months) was fixed, taking into account the yearly proportion of veal production in each slaughterhouse. The percentage of calves in the sample was set at the national level at 25%, bearing in mind that it was estimated at around 30% of French production in heads at slaughterhouses in 2007 (around 15% in tons).

#### Imported samples

Sampling was performed at the International Food Market (IFM) of Rungis (Ile-de-France [IDF] region), which is the largest food market for trading (national/international) of agro-food products (fruits, vegetables, dairy and other animal products, seafood, etc.) in France. The sampling plan enabled a representative allocation between the sources of imported carcasses, while covering 99% of the origins of the bovines, with a minimum of 30 analyses per country of origin.

### Sampling protocol

All samples were collected between January and July 2009. For the samples of French origin, only the heart was collected, while for the imported carcasses only the diaphragm was collected. An animal less than 8 months old was considered a calf, while an animal older than 8 months was considered an adult.

#### Samples of French origin

Fifty fresh hearts were collected per slaughterhouse and two slaughterhouses were sampled weekly. In the selected slaughterhouses, systematic random sampling was applied. The pace of sampling between two carcasses was defined by the ratio of the number of animals to be slaughtered that week/50 (animals to be sampled in 1 week). This method was designed to reduce the over-representation of animals raised together [13]. Hearts were collected in individual plastic boxes and kept refrigerated (+4 °C) for maximum 3 days before being sent to the National Reference Centre for toxoplasmosis in Reims (NRC, Reims, France) or to the National Reference Laboratory for food-borne parasites in Maisons-Alfort (NRL, Maisons-Alfort, France). Each laboratory received 50 fresh heart samples weekly, originating from only one slaughterhouse, according to the schedule plan.

Data about age and birth origin were collected for each sampled animal.

#### Imported samples

No hearts were available for the imported bovines, since they arrive in France as eviscerated carcasses. Therefore, the diaphragm was the only available sample, collected monthly and stored at −20 °C before being sent to the NRL (Maisons-Alfort, France).

### Serological tests

The modified agglutination test (MAT) for the detection of *T. gondii*-specific immunoglobulin (immunoglobulin G) antibodies was performed, as previously described, on all cardiac and diaphragm fluids using an antigen prepared from formalin-fixed whole RH tachyzoites produced and quality-controlled by the NRC (Reims, France) [33]. Cardiac fluids were collected by pipetting the exudate on the bottom of each plastic box. Meat juice was obtained from 25 g of diaphragms cut into small pieces and frozen overnight at −20 °C in a plastic bag. After thawing at room temperature, the meat juice was collected with a pipette into a microtube, as previously described [46]. The fluids were serially two-fold diluted. The threshold dilution was 1:6.

### Bioassay for *T. gondii* in mice

The *in vivo* experiments were approved by the local Animal Research Ethics Committee (ComEth Anses, EnvA, UPE) of Maisons-Alfort. In order to limit suffering and distress, mice were acclimated for 7 days after their arrival. Cages were filled with paper strips. Animal health and behaviour were monitored daily. Mice were observed based on the following criteria:

- external physical appearance (disheveled or spiked hairs, watering eyes, bent back, tremors),
- behaviour (exploratory behaviour decrease, unusual posture, prostration),
- behavioural response to external stimuli (no response).

If any of these criteria were critically altered, mice were subsequently euthanised and examined post-mortem to investigate the parasitic load. Euthanasia consisted in CO_2_ asphyxiation, followed by cervical dislocation.

After approval from the local Animal Research Ethics Committee, between 5 and 11 heart samples, randomly chosen from among those with the highest titers of agglutinating antibodies, were bioassayed weekly in three outbred female Swiss Webster mice (Charles River Laboratory, France). Additionally, nine seronegative hearts, randomly selected from the total number of samples, were bioassayed in mice. Briefly, each whole heart was mixed and incubated at 37 °C for 1.5 h with trypsin (final concentration 0.25%). The suspension was then filtered, pelleted by centrifugation, washed in saline, and resuspended in a saline solution containing penicillin G, streptomycin and amoxicillin to limit bacterial proliferation. This homogenate was inoculated intraperitoneally into three mice [1, 59]. Mice were monitored twice daily with food and water supplied *ad libitum*. In case of acute toxoplasmosis, the mice were culled by CO_2_ asphyxiation, followed by cervical dislocation, and samples of brain were taken for analysis. Mice were bled 4 weeks post-inoculation and their serum was tested at 1:25 dilution for *T. gondii* antibodies with the MAT. Later on, mice were culled 60 days post-inoculation by cervical dislocation, and their brains were examined for tissue cysts.

### Genotyping of *T. gondii* isolates

Brain cysts from seropositive mice were isolated by percoll gradient centrifugation [15]. DNA was extracted using a QIAamp DNA MiniKit (Qiagen, Courtaboeuf, France), and genotyping analysis of *T. gondii* DNA was performed with 15 microsatellite markers in a single multiplex PCR assay, as described elsewhere [3]. All strains isolated were cell cultivated and banked in the *Toxoplasma* Biological Resource Center (BRC, Reims).

### Statistical analysis

All analyses were performed in R (version 3.5.1), using the survey package (version 3.34) to fit the complex survey sample [42, 43]. Individual data were weighted for unequal sampling probability. Post-stratification was made in order to have two slaughterhouses and a minimum of 100 animals by geographical area. The complex design of the survey, considering the slaughterhouse as a cluster, and the geographical area as strata was taken into account in the statistical analysis and seroprevalence estimates. Imports were assumed to represent 15% of production. Imports were considered in French national seroprevalence estimates of beef, even though the tissue used for analysis was different (diaphragm), in order to obtain an overall estimate of exposure in France. Estimates of the differences in sensitivity or specificity between the two tissues (heart/diaphragm) used for autochthonous samples or imports were not available at the time of analysis, and as a consequence, these differences were not taken into account. Descriptive analyses were done for different dilutions in MAT at 1:6 to 1:200.

For French production, we analysed the probability of being seropositive with different explanatory variables such as the influence of age, sex, type of production (milk or meat), area of birth, life and slaughtering. Univariate analyses for the variable “age” (in the category “adult” or calf) and the variable “origin” (in the category “import” or “France”) were done using the Wald test.

For multivariate analysis, a sample showing a positive result in a 1:6 dilution in MAT was considered seropositive. The serological status was coded as 1 (positive) or 0 (negative). The response variable was analysed using logistic regression (quasi-binomial distribution and logit link function). The effect of age in years was also tested as a quantitative variable, with different polynomial degrees from linear regression to sixth degree polynomial regression. A first order interaction term was also tested. All possible models were compared using the lowest Akaike Information Criteria (AIC) [11]; AIC is defined using Rao-Scott approximation [44]. When several models show a difference in AIC less than 2, the model with the fewest number of parameters was retained, the most parsimonious one. The significance of each effect was determined using the Wald test.

## Results

### Sample collection

#### Samples of French origin

All 48 selected abattoirs were part of the sampling plan, except one (Vitré, Bretagne region). Therefore, two-fold increased sampling was carried out in one abattoir (Liffré, Bretagne region). A total of 2349 samples were collected; 574 of them were from calves and 1774 were from adults. For one sample, a qualitative indication of age (adult/calf) was not available. The area of birth was known for 1990 samples (359 unknown), of which 1473 were adults and 517 were calves. The minimum age at slaughtering in the dataset is 0.2 years, and the oldest animal was 20.4 years old. The mean age at slaughtering (taking into account the design of the survey), is 3.2 years, for a calf 5 and a half months (0.47 years), and for an adult 4.35 years old. Around 3% of observations (79/2349) were above or equal to 12 years old.

#### Imported samples

A total of 650 diaphragms were collected at the International Food Market (IFM) of Rungis (Ile-de-France (IDF) region). For 79 of them, no muscle juice was obtained due to an advanced condition of dehydration of the meat. Another seven were missing the origin country data. One was collected as an imported sample, but was in fact of French origin. A total of 563 animals were thus included in the statistical analysis, originating from Germany (143), the Netherlands (119), Ireland (84), Italy (71), Belgium (65), the United Kingdom (33), Austria (27), Poland (13), and other European countries: Denmark (1), Lithuania (4), Latvia (1), the Czech Republic (1) and Slovakia (1), representing 90.7% of the relative frequency in French meat imports (Table 7). Moreover, 225 were samples from calves and 338 from adults, without a precise indication of the age (in years or months).

### Serological tests

The results of the MAT according to the origin of samples (French/imported), age of animals (calves/adults), and terminal titer are presented in Table 1. In total, there were 318, 124, 117, 16, 12, 10 and 1 sample positive at a terminal titer of 6, 10, 25, 50, 100, 200 and 400, respectively.

**Table 1:** Results of the modified agglutination test (MAT) accordingly to the origin of samples (French/imported), age of animals (calves/adults) and terminal titer.

	Number of examined samples	Number of samples with terminal titer of:							
		0	6	10	25	50	100	200	400
Samples of French origin									
Calves	574	535	25	6	4	3	1	0	0
Adults	1774	1334	266	90	52	12	11	8	1
Imported samples									
Calves	225	211	3	3	8	0	0	0	0
Adults	338	233	24	25	53	1	0	2	0
Total	2912*	2314*	318	124	117	16	12	10	1

* For French samples, one information is missing (adults/veal), one is added to total.

### Seroprevalence

#### Overall seroprevalence

The overall seroprevalence in beef consumed in France (imports included) was found to be 17.38% [12.73–23.26] at a cut-off titer of 6. A decrease was observed when a higher cut-off was defined: at a titer of 10, the overall seroprevalence was 8.93 [5.97–13.17] and at a titer of 25, the overall seroprevalence was 5.01 [2.73–9.02] (Table 2).

**Table 2:** The overall seroprevalence of *Toxoplasma gondii* infection in beef consumed in France accordingly to the terminal titer (6; 10; 25; 50; 100; 200). Only one positive sample at terminal titer of 400 (adult of French origin).

All Mean and 95% CI	6	10	25	50	100	200
Samples of French origin (*N* = 2349*)	16.96 [11.91–23.56]	7.77 [4.91–12.09]	3.93 [1.90–7.95]	1.58 [0.54–4.52]	0.96 [0.30–3.05]	0.52 [0.12–2.17]
Imported samples (*N* = 563)	19.75 [7.88–41.48]	15.52 [6.32–33.32]	11.15 [3.54–30.04]	0.57 [0.07–4.33]	0.38 [0.05–2.91]	0.38 [0.05–2.91]
Overall (*N* = 2912*)	17.38 [12.73–23.26]	8.93 [5.97–13.17]	5.01 [2.73–9.02]	1.43 [0.53–3.81]	0.87 [0.30–2.49]	0.50 [0.14–01.74]

*Note*. % prevalence, 95% CI: 95% confidence interval of prevalence.

* One information missing (adults/veal), one is added to total.

The seroprevalence for calves was estimated to be 5.34% [2.49–11.09] at a cut-off titer of 6 (Table 3), and for adults 23.12% [17.83–29.40] at a cut-off titer of 6 (Table 4). As found previously, the seroprevalence decreased for both categories when a higher cut-off was chosen. Age as a qualitative variable (calves/adults) was found to be highly significant in univariate analysis (*p* significance or *p*-value = 2 × 10^−5^).

**Table 3:** The overall seroprevalence of *Toxoplasma gondii* infection in calves consumed in France accordingly to the terminal titer (6; 10; 25; 50; 100; 200).

All Mean and 95% CI	6	10	25	50	100	200
Samples of French origin (*N* = 574)	5.06 [1.79–13.50]	2.44 [0.35–14.94]	1.27 [0.17–8.89]	0.61 [0.08–4.68]	0.01 [0.00-0.12]	0
Imported samples (*N* = 225)	6.34 [4.94–8.10]	5.17 [3.28–8.06]	3.67 [1.59–8.22]	0	0	0
Overall (*N* = 799)	5.34 [2.49–11.09]	3.03 [0.89–9.82]	1.78 [0.56–5.51]	0.48 [0.06–3.55]	0.01 [0.00–0.09]	0

**Table 4:** The overall seroprevalence of *Toxoplasma gondii* infection in adults consumed in France accordingly to the terminal titer (6; 10; 25; 50; 100; 200).

All Mean and 95% CI	6	10	25	50	100	200
Samples of French origin (*N* = 1774)	22.00 [16.16–29.22]	10.03 [7.22–13.79]	5.05 [2.76–9.07]	1.99 [0.76–5.10]	1.36 [0.42–4.34]	0.74 [0.17–3.11]
Imported samples (*N* = 338)	31.41 [19.23–46.83]	24.51 [15.38–36.72]	17.65 [7.51–36.14]	1.07 [0.20–5.51]	0.71 [0.13–3.71]	0.71 [0.13–3.71]*
Overall (*N* = 2112)	23.12 [17.83–29.40]	11.75 [8.51–16.00]	6.55 [3.78–11.12]	1.88 [0.78–4.46]	1.28 [0.45–3.64]	0.73 [0.21–2.54]

* No terminal titer at dilution 100 (but at 200 or 50).

#### Samples of French origin

The overall seroprevalence of *Toxoplasma gondii* in cattle slaughtered in France was estimated at a cut-off titer of 6 to be 16.96% [11.91–23.56] (Table 2), with a specific seroprevalence of 5.06% [1.79–13.50%] for calves (Table 3) and 22.00% [16.16–29.22%] for adults (Table 4). As found previously, the seroprevalence decreased for both categories, when a higher cut-off was chosen. The effect of age as a qualitative variable (calves/adults) in univariate analysis was found to be highly significant (*p* = 0.00081).

**Figure 2:**
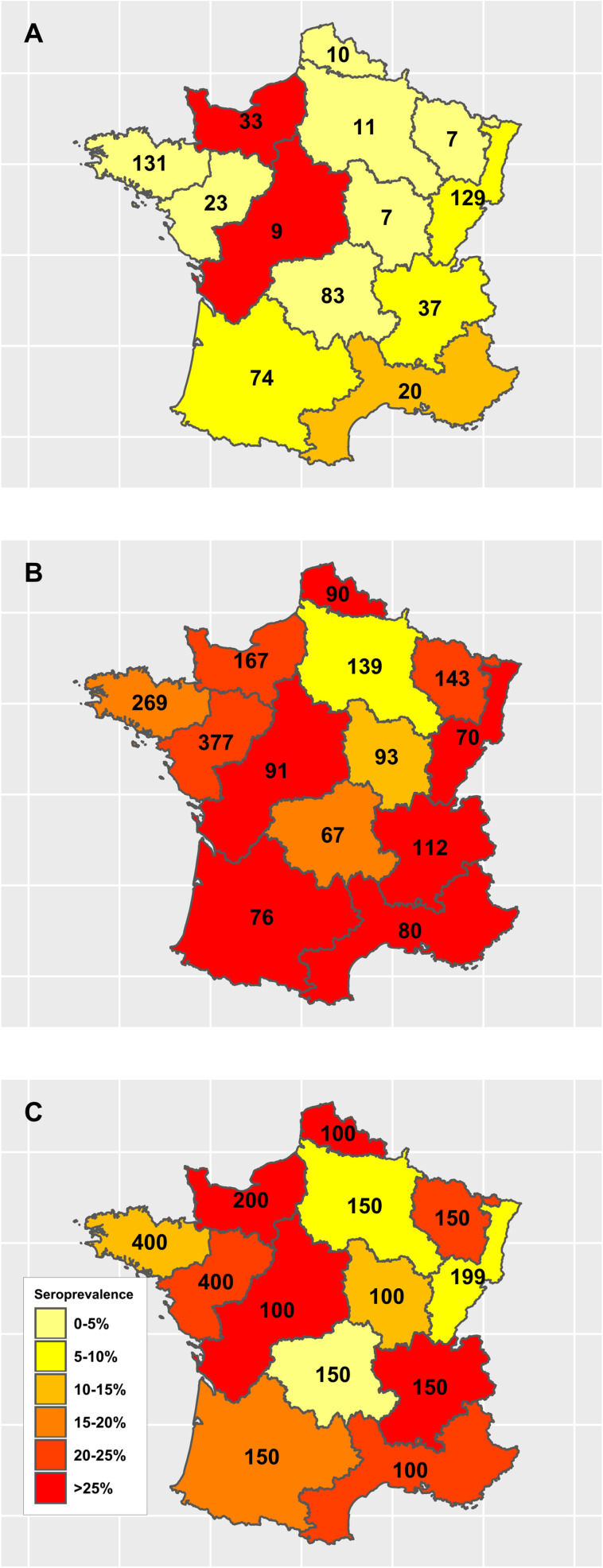
Geographical variation of *Toxoplasma gondii* seroprevalence of French bovine samples according to the area of slaughtering and to age categories: (A) calves; (B) adults; (C) bovines overall (calves and adults). The numbers represent the number of samples collected for each region.

Depending on to the region of slaughter, seroprevalence varied between 0% and 55.56% for calves and from 5.95% to 35.31% for adults (Table 5), with no specific geographical pattern identified (Fig. 2A–C). The seroprevalence estimates were markedly lower in calves than in adults, in all regions, except Centre, Poitou-Charentes and Normandie. Seroprevalence estimates by areas of birth are also given in Table 6, with the same identified difference between calves and adults, which stands for all regions, except IDF, Picardie, and Champagne-Ardennes.

**Table 5:** Seroprevalence of *Toxoplasma gondii* infection (cut-off titer 6) in bovine meat consumed in France by area of slaughtering.

Area of slaughtering	Bovine (adults and calves)		Adults		Calves	
	% (No. of animals sampled)	95% CI	% (No. of animals sampled)	95% CI	% (No. of animals sampled)	95% CI
Alsace-Franche Comté	9.92 (199)	3.01–28.08	33.53 (70)	14.88–59.29	5.61 (129)	2.01–14.68
Aquitaine Midi-Pyrénées	16.02 (150)	13.14–19.38	25.28 (76)	15.16–39.04	7.27 (74)	4.08–12.63
Auvergne-Limousin	3.64 (150)	0.51–21.70	15.60 (67)	7.77–28.87	0 (83)	0
Bourgogne	12.59 (100)	8.05–19.16	13.39 (93)	9.52–18.51	0 (7)	0
Bretagne	12.01 (400)	6.91–20.07	16.84 (269)	10.67–25.57	0 (131)	0
Centre-Poitou Charentes	30.79 (100)	11.58–60.20	29.65 (91)	11.51–57.71	55.56 (9)	21.12–85.37
IDF-Picardie-Champagne-Ardennes	5.61 (150)	2.86–10.71	5.95 (139)	3.10–11.15	1.49 (11)	0.11–17.10
Languedoc-Roussillon-PACA	23.33 (100)	17.35–30.61	27.93 (80)	17.22–41.93	15.00 (20)	4.92–37.59
Lorraine	22.49 (150)	8.17–48.60	23.66 (143)	8.89–49.62	0 (7)	0
Nord-Pas-de-Calais	27.48 (100)	19.17–37.72	30.98 (90)	23.82–39.19	0 (10)	0
Normandie	26.16 (200)	7.92–59.31	24.25 (167)	8.55–52.29	34.02 (33)	6.27–79.88
Pays de la Loire	21.19 (400)	9.65–40.35	22.66 (377)	10.39–42.53	0 (23)	0
Rhône-Alpes*	28.73 (150*)	8.10–64.82	35.31 (112)	9.99–72.86	5.94 (37)	1.48–20.97
Bovine meat consumed in France (French slaughterhouses)*	16.96 (2349*)	11.91–23.56	22.00 (1774)	16.16–29.22	5.06 (574)	1.79–13.50

*Note*. % prevalence, 95% CI: 95% confidence interval of prevalence.

* One information missing (adults/veal), one is added to total.

**Table 6:** Seroprevalence of *Toxoplasma gondii* infection (cut-off titer 6) in bovine meat consumed in France by area of birth.

Area of slaughtering	Bovine (adults and calves)		Adults		Calves	
	% (No. of animals sampled)	95% CI	% (No. of animals sampled)	95% CI	% (No. of animals sampled)	95% CI
Alsace-Franche Comté	12.38 (60)	7.04–20.86	14.25 (36)	8.62–22.65	7.81 (24)	1.45–32.85
Aquitaine Midi-Pyrénées	13.22 (121)	5.73–27.64	20.45 (63)	10.64–35.70	4.32 (58)	0.79–20.38
Auvergne-Limousin	16.38 (197)	8.34–29.67	30.55 (113)	17.45–47.79	2.96 (84)	0.94–8.91
Bourgogne	30.95 (111)	15.09–53.06	34.35 (99)	16.51–58.08	3.57 (12)	0.55–19.95
Bretagne	17.96 (206)	7.80–36.16	24.43 (126)	11.48–44.61	9.87 (80)	1.54–43.40
Centre-Poitou Charentes	14.02 (228)	6.97–26.20	24.51 (144)	13.99–39.34	4.51 (84)	1.34–14.11
IDF-Picardie-Champagne-Ardennes	5.40 (141)	2.01–13.71	5.15 (122)	1.54–15.86	6.47 (19)	1.44–24.65
Languedoc-Roussillon-PACA	17.85 (64)	11.23–27.19	30.78 (54)	15.25–52.35	3.74 (10)	0.40–27.17
Lorraine	3.84 (101)	0.62–20.36	4.25 (97)	0.67–22.63	0 (4)	0
Nord-Pas-de-Calais	23.80 (50)	17.62–31.32	29.96 (39)	25.44–34.90	1.05 (11)	0.11–9.10
Normandie	15.07 (249)	8.09–26.35	15.84 (228)	8.79–26.87	6.01 (21)	0.77–34.42
Pays de la Loire	20.07 (349)	10.95–33.91	25.24 (278)	14.28–40.62	4.26 (71)	0.95–17.14
Rhône-Alpes*	20.44 (113)	9.28–39.22	25.60 (74)	10.49–50.25	6.59 (39)	1.26–28.07
Bovine meat consumed in France (French slaughterhouses)*	16.96 (2349*)	11.91–23.56	22.00 (1774)	16.16–29.22	5.06 (574)	1.79–13.50

Note. % prevalence, 95% CI: 95% confidence interval of prevalence.

* One information missing (adults/veal), one is added to total.

**Figure 3:**
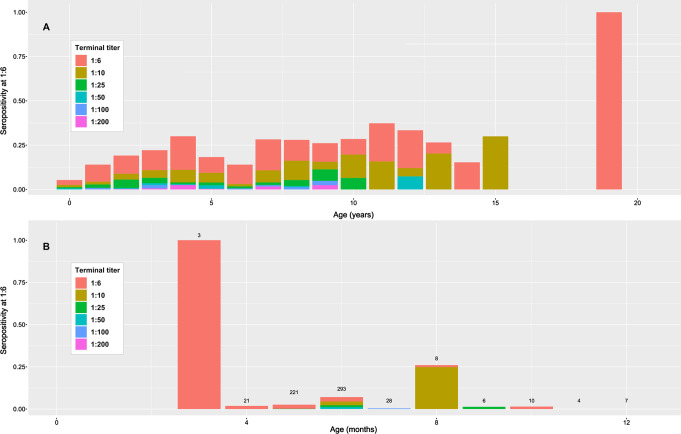
Terminal titer of the modified agglutination test (MAT) for French origin samples in relation to age (A) for all samples (*n* = 2348) (age in years); (B) only for bovines less than 1 year (*n* = 601) (age in months). The number of observations at each month of age is given at the top of the corresponding bar.

**Figure 4:**
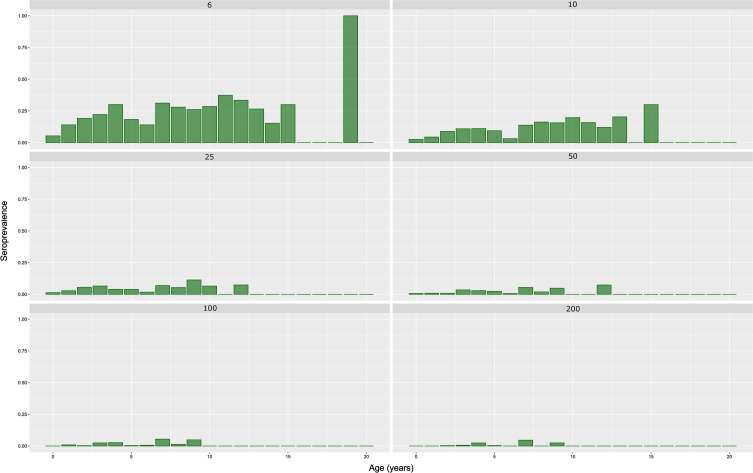
Seroprevalence of *Toxoplasma gondii* infection in bovines of French origin (adults + calves) accordingly to the age and the titer (6; 10; 25; 50; 100; 200).

The terminal titer of the MAT, in relation to age is presented in Figure 3 for all French samples (*n* = 2348) (age in years) (A) or only for bovines less than 1 year old (*n* = 601) (age in months) (B). No particular trend was observed, with higher or lower terminal titer specifically for young or old animals. The seroprevalence of *T. gondii* infection in bovines of French origin (adults + calves) according to the age and the titer (6; 10; 25; 50; 100; 200) is presented in Figure 4. The seroprevalence showed an increase in general at a younger age; however, after the age of 4–5 years, a general linear trend was not clearly observed.

#### Imported samples

The overall seroprevalence in imported beef at a cut-off titer of 6 was estimated to be 19.75% [7.88–41.48%] (Tables 2 and 7), with a distinct seroprevalence of 6.34% [4.94–8.10%] for calves (Tables 3 and 7) and 31.41% [19.23–46.83%] for adults (Tables 4 and 7). As found previously, the seroprevalence decreased for both categories, when a higher cut-off was chosen (Tables 2–4). The higher seroprevalence in imported animals compared to French origin bovines, for any of the chosen titers (Tables 2–4), was not found to be significant in univariate analysis, whether for bovines overall (adults + calves) (*p* = 0.70), or for adults (*p* = 0.13) or calves (0.65), respectively.

**Table 7:** Seroprevalence of *Toxoplasma gondii* infection (cut-off titer 6) in bovine meat imported at IFM Rungis (Ile de France).

Origin country	Relative frequency in French meat imports	Bovine (adults and calves)		Adults		Calves	
		% (No. of animals sampled)	95% CI	% (No. of animals sampled)	95% CI	% (No. of animals sampled)	95% CI
The Netherlands	25.1%	5.88 (119)	2.83–11.83	No data (0)	No data	5.88 (119)	2.83–11.83
Germany	24.7%	38.46 (143)	30.85–46.68	40.91 (132)	32.85–49.49	9.09 (11)	1.26–43.90
Ireland	15.3%	23.81 (84)	15.90–34.06	23.81 (84)	15.90–34.06	No data (0)	No data
Italy	10.1%	5.63 (71)	2.13–14.08	0 (1)	0	5.71 (70)	2.16–14.27
Belgium	8.4%	18.46 (65)	10.79–29.77	20.00 (60)	11.72 -32.01	0 (5)	0
Poland and other European countries*	4.8%	9.52 (21)	2.39–31.15	0 (1)	0	10.00 (20)	2.51–32.41
UK	1.5%	30.30 (33)	17.14–47.76	30.30 (33)	17.14–47.76	No data (0)	No data
Austria	0.8%	33.33 (27)	18.33–52.69	33.33(27)	18.33–52.69	No data (0)	No data
Total imports	90.7%	19.75 (563)	7.88–41.48	31.41 (338)	19.23–46.83	6.34 (225)	4.94–8.10

* Poland (13) + Denmark (1) + Lithuania (4) + Latvia (1) + Czech Republic (1) + Slovakia (1).

#### Risk factor analysis in samples of French origin

The analysis was restricted to animals below 12 years old since the number of observations above or equal to 12 years was 3% in the dataset, and in order to avoid fitting a nonlinear relationship with a small number of samples. The complete dataset for all explanatory variables, such as age, sex, type of production (milk or meat), area of birth, breeding and slaughtering, contained 1642 observations.

**Figure 5:**
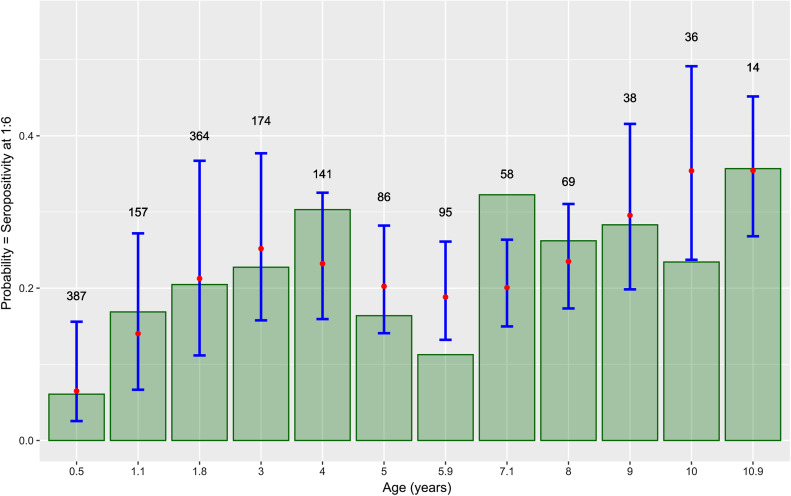
Comparison of observed values *versus* predicted values by the final model according to age. The observed values are in green bars, while for the predicted values the red point represents the mean prediction and the blue segment the 95% confidence interval of the prediction. The number above the blue segment is the number of observations for this particular class of age.

**Figure 6:**
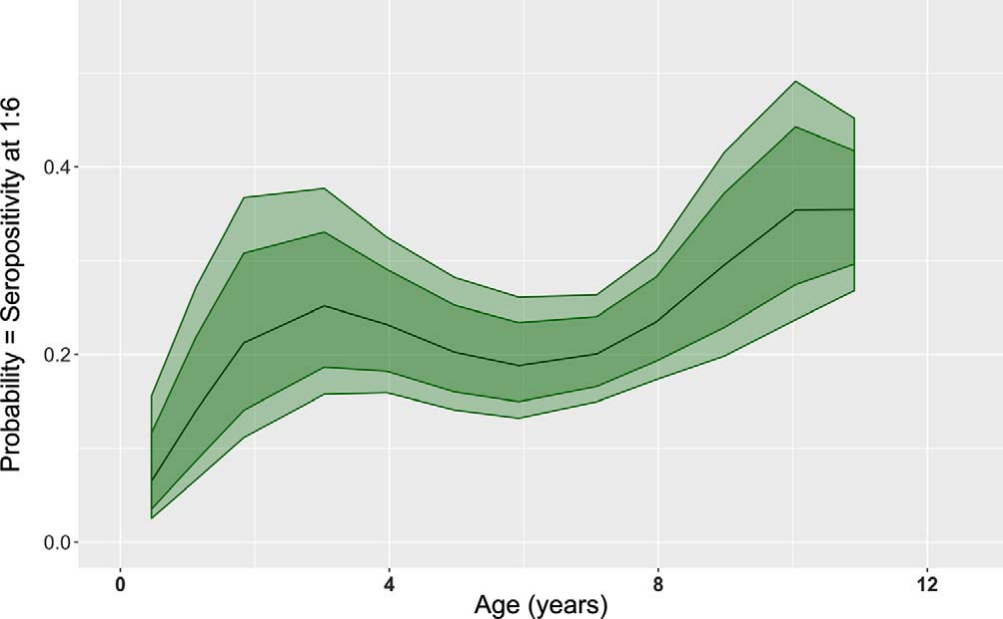
Prediction by the final model of the age-related seroprevalence (cut-off titer of 6), with the 95% confidence interval represented by the light green colour and the 80% confidence interval by the dark green colour.

In order to investigate different risk factors, 195 possible models were tested. The best model with the lowest Akaike’s Information Criterion (AIC = 1453.2) included only age as an explanatory variable. This final model is a polynomial model of degree 4 with age in years (Table 8), showing also that a linear model was not the best model. It is also the most parsimonious model for this range of AIC. Other models with differences in AIC lower than 2, included a polynomial of degree 4 for age in years and sex in interaction (AIC = 1453.66), and a polynomial of degree 5 for age in years (AIC = 1453.8). Figure 5 shows the relative agreement between observed values for age in class by year and the prediction of the final model for the mean age for each annual class. Except for the age class 6 (5.9 years in average) and 7 (7.1 years in average) years old, the final model fits the data: the seroprevalence for each particular age class, represented by the top of the grey bar, is inside the 95% confidence interval of the prediction of the model. Figure 6 shows the prediction of the final model, with a clear non-linear effect of age related seroprevalence (at a cut-off titer of 6).

**Table 8:** Final model to explain *Toxoplasma gondii* seropositivity at a cut-off titer of 6 (1642 observations).

Factor	Parameter estimate (Standard Error)	OR	95% CI OR	*P* value (Wald Test)
Intercept	−3.547 (0.657)	0.029	0.008–0.104	2.37 × 10^−5^
Age	2.190 (0.694)	8.931	2.293–34.783	0.0047
Age^2^	−0.659 (0.254)	0.517	0.315–0.851	0.0169
Age^3^	0.076 (0.034)	1.079	1.010–1.153	0.0340
Age^4^	−0.003 (0.001)	0.997	0.994–1	0.0551

### *Toxoplasma gondii* isolation and genotyping

The digestion products of 209 heart samples, obtained from both serologically positive (*n* = 200) and negative (*n* = 9) animals, were inoculated in mice. In total, 627 mice were used for the bioassay. For four bovine samples (8, 76, 122, 125 months; terminal titer of 10, 10, 25 and 10, respectively), all three mice died in the first 48 h post-inoculation (p.i)., yielding 205 bioassay results. For two samples, corresponding to serologically positive adult bovines (4.8 and 7 years old; terminal titer of 25 and 10), collected in Franche-Comté and Languedoc-Roussillon region slaughterhouses, we noted seroconversion in the inoculated mice 4 weeks p.i.; a microscope examination at 60 days p.i. confirmed the presence of tissue cysts in brains. Both strains were genotyped as type II with all 15 microsatellite markers (TUB2, W35, TgM-A, B18, B17, M33, MIV.1, MXI.1, M48, M102, N60, N82, AA, N61, N83) being amplified. They can be found in the *Toxoplasma* Biological Resource Center, Reims, France (toxocrb.com) under accession numbers: TgA 32127 and TgA 32128. No strains were isolated from the heart samples corresponding to the nine serologically negative bovines. Detailed data about the number of collected and bioassayed samples of French origin according to the terminal titer + and age can be found in the Supplementary file.

## Discussion

Following a previous study and using a sampling plan based on ovine meat production data and innovative testing for antibody detection and parasite isolation [33], the present survey, conducted according to the same principles of sampling and testing, aimed at investigating the national *T. gondii* seroprevalence in beef consumed in France. Therefore, the calculated seroprevalence should be considered the most representative value for *T. gondii* in beef in France, originating either from slaughtered or imported carcasses. Currently, France is one of the largest producers of beef in Europe, but the import of 300,000 tons per year remains necessary [12]. Compared to what was initially planned for the collected samples from imports, several countries were absent, in particular Spain and Brazil, representing 7.5% and 0.8% of the relative frequency in French meat imports. This explains the 90.7% of the total imports, which represents an acceptable level of sample collection.

The overall seroprevalence of *T. gondii* in bovine carcasses was 17.38% [12.73–23.26] at a cut-off titer of 6. This is in accordance with previous results obtained in France [31, 52], in other European countries (Spain: 18.6% [4], Estonia 18.62% [38]), or elsewhere in the world (Senegal: 13% [17]), with the same technique and testing a high number of samples (>1000). However, seroprevalence in cattle varies greatly throughout the world, ranging from 0% to 92% [2, 10, 21, 26, 31, 36–41, 45, 47, 50, 51, 55, 57]. This variation is due to a large number of factors, such as climate, herd-management, cat population, sampling procedure, or diagnosis tools [31, 40, 47]. Therefore, in order to introduce less bias for comparison between studies, we used the MAT technique, which has already been demonstrated to be superior to other tests (Sabin-Feldman Dye Test, latex agglutination, indirect haemagglutination) for serological screening of cattle [22]. The choice of a low threshold (cut-off titer of 6) was justified in our case by (a) our goal: to isolate viable *T. gondii* parasites, considering at that time that viable parasites should mostly be present in seropositive animals; (b) the fact that generally in bovines one can find very low titers, in our case the highest titer was observed in only one bovine at a terminal titer of 400, most of the animals (559/598) having a titer lower than 50; and (c) the decision to work on muscle (cardiac/diaphragm) fluid, representing a low-concentration antibody matrix compared to serum. Therefore our titer corresponds to a higher titer in blood sera. A 10-fold equivalence between the two matrices has been suggested in a study comparing meat juice and serum for *Trichinella* detection [46].

A strong age (adult/calves) effect was observed with a seroprevalence of 5.34% [2.49–11.09] for calves (animals less than 8 months of age) and 23.12% [17.83–29.40] for adults (animals over 8 months of age) at a cut-off titer of 6. The age difference was valid for both types of samples: French and imported, and for all French regions except Normandie and Centre-Poitou Charentes, where a higher seroprevalence was observed in calves than in adults. However, regarding confidence intervals this difference was not meaningful. This difference could be speculated to be due to the low number of animals (33 and 9, respectively) or to a common source of infection. Differences in seroprevalence between calves and adults may be related to the oral route of infection, older animals being exposed to the parasite for a longer period of time than younger ones. The overall seroprevalence and the significant age difference in the seroprevalence in cattle, follows the same pattern that has already been observed in France for other meat-producing animals [19, 33]. Nevertheless, the percentages were significantly higher for other ruminants (sheep), both for adult meat (81%) and lamb (15%).

The collection of French samples, and their associated data, enabled us to analyse the effect of several parameters: age and sex of animals, geographical origin and bovine meat production level in the area where the sample was collected. Other parameters such as seasonal variations, herd characteristics and herd management were not taken into account because of several reasons: the timeframe was limited (6-month period); the goal of our study was to estimate seroprevalence of *T. gondii* infection in fresh beef consumed in France and not in the cattle population of France. Nevertheless, statistical analyses reveal that the major factor to explain variations in the seroprevalence observed is the age of the animal at a threshold dilution of 1:6 with a non-linear relationship (polynomial of degree 4). A non-linear relationship was also found graphically between age and seroprevalence at any cut-off (Figs. 4–6), suggesting no life-long immunity and possible re-infection by *T. gondii* in cattle. Similar age-related seroprevalence patterns were obtained in a recent study in Estonia [38], where the seroprevalence increased until the age of 5 years (60–71 months), after which there was no evident linear pattern. One possible explanation might be that antibodies do not persist in cattle, already shown by Dubey and Thulliez [26] and Gilot-Fromont et al. [31], while antibodies could persist several years in sheep [27], pigs [19], or other animals [28]. Another explanation might be that seropositive animals were culled earlier, therefore *T. gondii* infection might be negatively associated with longevity [38]. Moreover, it was shown that the presence of cats modified the age-seroprevalence relationship [31]. This factor could not be tested here, and our population probably mixed animals with life-history presence of cats and absence of cats. At the same time, no clear relationship was detected between the terminal titer and age, with calves having low titers, adults having high titers, or the other way around (Fig. 3A and B). In our population, the high titers (>50) were found in animals less than 10 years old, together also with low titers, while in older animals (>10 years), we only found low titers (<50). To our knowledge, no other recent study has investigated this pattern, so only speculations about the loss of immunity in older cattle might explain this, as previously mentioned [26, 31]. Concerning the animals younger than 1 year, one can clearly see that calves around 3–4 months are positive at a low titer (6). However, this result was for a small number of animals, while animals between 6 and 9 months were found to be positive at various titers (high and low), suggesting the detection/presence of maternal antibodies (3–4 months) and the first *T. gondii* infection (6–9 months), with the decrease of maternal immunity. Similar situations with the decrease of maternal antibodies and the first natural *T. gondii* infection revealed by higher antibodies titers are found in other animal species, such as sheep [60] or pigs [19]. When comparing the seroprevalence estimates given by area of slaughtering (Table 5) and area of birth (Table 6), a variation was observed, showing that the areas with the highest seroprevalence were not the same between these two variables. This is true for adults and bovines overall (calves + adults), while for calves the number of positive analyses was too low to continue the comparison. One of the hypotheses to explain this variation is the commercial trade: a similar seroprevalence between birth and slaughtered area should be found only at a local level, not in the case of a national survey, when samples are collected from animals that are potentially born in region A, raised in region B, and slaughtered in region C. This hypothesis is also supported by a recent UK study [37], showing that the number of sites an animal stayed before slaughter ranged from 1 to 15 (median 3), thereby multiplying the possibilities of *T. gondii* oocyst ingestion, and making it relatively impossible to identify the infection site. A similar situation might also be present in France. Moreover, the age of slaughtering can be seen as a confounding factor for comparing different areas.

Our study enabled us to observe that several regions (Aquitaine, Midi Pyrénées, Nord-Pas de Calais), for which the seroprevalence was high in adult animals, and that provide meat that is eaten raw or undercooked (*bleu* or *saignant*) (Fig. 2B), corresponded to those for which seroprevalence was also high in humans [10, 58]. Our results are in agreement with the previous study of Halos et al. [33], that found similar areas of concordance/correlation between seroprevalence of *T. gondii* in humans and lamb, the lamb representing the other type of meat that is often eaten undercooked (*agneau rosé*) in France. No direct link can yet be made between the presence of *T. gondii* in meat-producing animals and human infection, except if we assume that locally produced meat is consumed locally (very unlikely). On the other hand, other regions with high seroprevalence in adult animals (Rhone-Alpes, Centre-Poitou Charentes) do not present a high seroprevalence in humans. Therefore, the agreement between *T. gondii* seroprevalence in humans and cattle in several areas of France should be interpreted carefully, as it remains unexplained. It could be speculated to be due to more intense circulation of the parasite in certain areas, but this requires further investigation.

Concerning the bioassays and parasite isolation, several parameters need to be taken into account: first, only a limited number of samples (209/2349) could be tested, thus reducing our analysis on the prevalence of viable *T. gondii* tissue cysts in beef consumed in France. Second, bioassays in mice are less sensitive than those in cats [24], especially in cases where the whole heart digest could not be fully inoculated. Third, our results probably underestimate the effective parasitological prevalence in beef, since we mostly tested serologically positive animals (200/209). This was done based at that time on the results from the ovine survey [33], where the chance of obtaining viable parasites increased with the titer and age. Meanwhile, in a study on a limited number of bovines (*n* = 100), the authors identified two PCR-positive results, while the corresponding MAT results were negative (cut-off 1:40). Similarly, three MAT-positive results had corresponding negative PCR results [49]. In another recent study on 167 calves and 235 adults, the authors demonstrated that serological testing by MAT or p30 immunoblot does not provide reliable information about the presence of *T. gondii* parasites or DNA in cattle [50]. In our case, however, two strains (1%) were isolated from heart samples of low titer MAT-positive cattle, adding to the limited number of such reports [7, 25, 50, 51]. The presence of live parasites in the heart of naturally infected cattle does not indicate their presence in other parts of carcasses that will be consumed. Nevertheless, previous studies showed persistence of viable *T. gondii* in tissues likely to be eaten, including the heart, after experimental infection with oocysts [26]. Accurate knowledge of the distribution of the parasite within cattle carcasses is still needed [16, 18, 28, 29]. Similar results have previously been obtained for the ovine meat consumed in France, with a significantly higher prevalence of parasite isolation (5.4%) from fresh carcasses [33].

The animals in this study were sampled in 2009, and it should be emphasized that the epidemiological situation may have changed since then. Nevertheless, the samples were collected and analysed in a comparative way with the national surveys of sheep and pigs [19, 33]. Therefore, the present study adds to knowledge on the presence of *T. gondii* in the three main meat producing animals in France, offering new and reliable facts about *T. gondii* presence in bovines. Overall, our results indicate that there is a risk of human infection with *T. gondii* following beef consumption in France, which still needs to be correlated with the food habit of ingesting raw/undercook (*bleu* or *saignant*) beef [9, 58]. However, new questions have emerged specifically concerning the isolation of parasites from beef and the precise role of bovines, generally described as poor hosts for *T. gondii*, in human infection. Similar surveys should be conducted in other countries, to obtain a broader view of animal sources of *T. gondii* infection in humans.

## Supplementary materials

Table. Number of collected/bioassayed samples of French origin, according to the terminal titer and the age of animals. Click here for additional data file. Supplementary material is available at https://www.parasite-journal.org/10.1051/parasite/2019076/olm.

## References

[1] Afonso E, Poulle ML, Lemoine M, Villena I, Aubert D, Gilot-Fromont E. 2007 Prevalence of *Toxoplasma gondii* in small mammals from the Ardennes region, France. Folia Parasitologica, 54, 313–314. 18303774 10.14411/fp.2007.041

[2] AFSSA. 2005 Toxoplasmose : état des connaissances et évaluation du risque lié à l’alimentation Rapport du groupe de travail “*Toxoplasma gondii*” de l’Afssa. p. 318.

[3] Ajzenberg D, Collinet F, Mercier A, Vignoles P, Darde ML. 2010 Genotyping of *Toxoplasma gondii* isolates with 15 microsatellite markers in a single multiplex PCR assay. Journal of Clinical Microbiology, 48(12), 4641–4645. 20881166 10.1128/JCM.01152-10 PMC3008440

[4] Almería S, Cabezón O, Paniagua J, Cano-Terriza D, Jiménez-Ruiz S, Arenas-Montes A, Dubey JP, García-Bocanegra I. 2018 *Toxoplasma gondii* in sympatric domestic and wild ungulates in the Mediterranean ecosystem. Parasitology Research, 117(3), 665–671. 29344801 10.1007/s00436-017-5705-6

[5] Aspinall TV, Marlee D, Hyde JE, Sims PF. 2002 Prevalence of *Toxoplasma gondii* in commercial meat products as monitored by polymerase chain reaction – food for thought? International Journal for Parasitology, 32(9), 1193–1199. 12117502 10.1016/s0020-7519(02)00070-x

[6] Baril L, Ancelle T, Goulet V, Thulliez P, Tirard-Fleury V, Carme B. 1999 Fisk factors for toxoplasma infection in pregnancy: a case-control study in France. Scandinavian Journal of Infectious Diseases, 31, 305–309. 10482062 10.1080/00365549950163626

[7] Belluco S, Mancin M, Conficoni D, Simonato G, Pietrobelli M, Ricci A. 2016 Investigating the determinants of *Toxoplasma gondii* prevalence in meat: a systematic review and meta-regression. PLoS One, 11(4), e0153856. 27082633 10.1371/journal.pone.0153856 PMC4833317

[8] Belluco S, Patuzzi I, Ricci A. 2018 Bovine meat versus pork in *Toxoplasma gondii* transmission in Italy: a quantitative risk assessment model. International Journal of Food Microbiology, 269, 1–11. 29358131 10.1016/j.ijfoodmicro.2017.12.026

[9] Berger F, Goulet V, Le Strat Y, Desenclos JC. 2009 Toxoplasmosis among pregnant women in France: risk factors and change of prevalence between 1995 and 2003. Revue Épidemiologique de Santé Publique, 57(4), 241–248. 10.1016/j.respe.2009.03.006 19577390

[10] Berger-Schoch AE, Bernet D, Doherr MG, Gottstein B, Frey CF. 2011 *Toxoplasma gondii* in Switzerland: a serosurvey based on meat juice analysis of slaughtered pigs, wild boar, sheep and cattle. Zoonoses Public Health, 58(7), 472–478. 21824348 10.1111/j.1863-2378.2011.01395.x

[11] Burnham KP, Anderson DR. 2002 Model selection and multimodel inference: a practical information-theoretic approach, 2nd edn New York: Springer-Verlag p. 488.

[12] Chatellier V. 2017 Les échanges de bovins vivants et de viande bovine dans le monde et dans l’UE : trajectoires productives et commerciales des principaux pays impliqués. INRA Productions Animales, 30(3), 19.

[13] Cochran WG. 1977 Sampling techniques. Wiley series in probability and mathematical statistics-applied. New York: Wiley p. 413.

[14] Cook AJ, Gilbert RE, Buffolano W, Zufferey J, Petersen E, Jenum PA, Foulon W, Semprini AE, Dunn DT. 2000 Sources of toxoplasma infection in pregnant women: European multicentre case-control study. European Research Network on Congenital Toxoplasmosis. British Medical Journal, 321(7254), 142–147. 10894691 10.1136/bmj.321.7254.142 PMC27431

[15] Cornelissen AW, Overdulve JP, Hoenderboom JM. 1981 Separation of *Isospora* (*Toxoplasma*) *gondii* cysts and cystozoites from mouse brain tissue by continuous density-gradient centrifugation. Parasitology, 83, 103–108. 6267543 10.1017/s0031182000050071

[16] Costa GH, da Costa AJ, Lopes WD, Bresciani KD, dos Santos TR, Esper CR, Santana AE. 2011 *Toxoplasma gondii*: infection natural congenital in cattle and an experimental inoculation of gestating cows with oocysts. Experimental Parasitology, 127(1), 277–281. 20736009 10.1016/j.exppara.2010.08.005

[17] Davoust B, Mediannikov O, Roqueplo C, Perret C, Demoncheaux JP, Sambou M, Guillot J, Blaga R. 2015 Serological survey of animal toxoplasmosis in Senegal. Bulletin de la Société de Pathologie Exotique, 108(1), 73–77. 25307881 10.1007/s13149-014-0403-4

[18] de Macedo MF, de Macedo CA, Ewald MP, Martins GF, Zulpo DL, da Cunha IA, Taroda A, Cardim ST, Su C, Garcia JL. 2012 Isolation and genotyping of *Toxoplasma gondii* from pregnant dairy cows (Bos taurus) slaughtered. Revista Brasileira de Parasitologia Veterinária, 21(1), 74–77. 22534951 10.1590/s1984-29612012000100016

[19] Djokic V, Blaga R, Aubert D, Durand B, Perret C, Geers R, Ducry T, Vallee I, Djurkovic Djakovic O, Mzabi A, Villena I, Boireau P. 2016 *Toxoplasma gondii* infection in pork produced in France. Parasitology, 143(5), 557–567. 26928081 10.1017/S0031182015001870

[20] Dohoo I, Martin W, Stryhn H. 2003 Veterinary epidemiologic research. Charlottetown, Canada: Atlantic Veterinary College p. 521–539.

[21] Dong H, Lu YY, Su RJ, Wang YH, Wang MY, Jiang YB, Yang YR. 2018 Low prevalence of antibodies against *Toxoplasma gondii* in dairy cattle from China’s central region. BMC Veterinary Research, 14(1), 315. 30340586 10.1186/s12917-018-1629-3 PMC6194598

[22] Dubey JP, Desmonts G, McDonald C, Walls KW. 1985 Serologic evaluation of cattle inoculated with *Toxoplasma gondii*: comparison of Sabin-Feldman dye test and other agglutination tests. American Journal of Veterinary Research, 46(5), 1085–1088. 4003883

[23] Dubey JP. 1986 A review of toxoplasmosis in cattle. Veterinary Parasitology, 22(3–4), 177–202. 3551316 10.1016/0304-4017(86)90106-8

[24] Dubey J, Beattie C. 1988 Toxoplasmosis in man (*Homo sapiens*), in Toxoplasmosis of animals and man, Dubey J, Beattie C, Editors. CRC Press: Florida p. 41–60.

[25] Dubey JP. 1992 Isolation of *Toxoplasma gondii* from a naturally infected beef cow. Journal of Parasitology, 78(1), 151–153. 1738059

[26] Dubey JP, Thulliez P. 1993 Persistence of tissue cysts in edible tissues of cattle fed *Toxoplasma gondii* oocysts. American Journal of Veterinary Research, 54(2), 270–273. 8430937

[27] Dubey JP. 2009 Toxoplasmosis in sheep – the last 20 years. Veterinary Parasitology, 163, 1–14. 19395175 10.1016/j.vetpar.2009.02.026

[28] Dubey JP. 2010 Toxoplasmosis of animals and humans, 2nd ed, Boca Raton, FL: CRC Press p. 336.

[29] Esteban-Redondo I, Maley SW, Thomson K, Nicoll S, Wright S, Buxton D, Innes EA. 1999 Detection of *Toxoplasma gondii* in tissues of sheep and cattle following oral infection. Veterinary Parasitology, 86(3), 155–171. 10511098 10.1016/s0304-4017(99)00138-7

[30] France Agri Mer. 2015 Impact de la crise économique sur la consommation de viandes et évolutions des comportements alimentaires. Les synthèses de France Agri Mer. p. 21.

[31] Gilot-Fromont E, Aubert D, Belkilani S, Hermitte P, Gibout O, Geers R, Villena I. 2009 Landscape, herd management and within-herd seroprevalence of *Toxoplasma gondii* in beef cattle herds from Champagne-Ardenne, France. Veterinary Parasitology, 161(1–2), 36–40. 19155137 10.1016/j.vetpar.2008.12.004

[32] Gottstein B, Hentrich B, Wyss R, Thur B, Busato A, Stark KD, Muller N. 1998 Molecular and immunodiagnostic investigations on bovine neosporosis in Switzerland. International Journal for Parasitology, 28(4), 679–691. 9602392 10.1016/S0020-7519(98)00006-X PMC7130244

[33] Halos L, Thebault A, Aubert D, Thomas M, Perret C, Geers R, Alliot A, Escotte-Binet S, Ajzenberg D, Darde ML, Durand B, Boireau P, Villena I. 2010 An innovative survey underlining the significant level of contamination by *Toxoplasma gondii* of ovine meat consumed in France. International Journal for Parasitology, 40(2), 193–200. 19631651 10.1016/j.ijpara.2009.06.009

[34] Hayde M, Pollac AF. 2000 Clinical picture. Neonatal signs and symptoms, in . in Congenital toxoplasmosis, Ambroise-Thomas P, Pedersen E, Editors Springer-Verlag: Paris p. 153–164.

[35] Hill D, Coss C, Dubey JP, Wroblewski K, Sautter M, Hosten T, Munoz-Zanzi C, Mui E, Withers S, Boyer K, Hermes G, Coyne J, Jagdis F, Burnett A, McLeod P, Morton H, Robinson D, McLeod R. 2011 Identification of a sporozoite-specific antigen from *Toxoplasma gondii*. Journal of Parasitology, 97(2), 328–337. 21506817 10.1645/GE-2782.1 PMC3684278

[36] Holec-Gąsior L, Drapała D, Dominiak-Górski B, Kur J. 2013 Epidemiological study of *Toxoplasma gondii* infection among cattle in Northern Poland. Annals of Agricultural and Environmental Medicine, 20(4), 653–656. 24364429

[37] Hosein S, Limon G, Dadios N, Guitian J, Blake DP. 2016 *Toxoplasma gondii* detection in cattle: a slaughterhouse survey. Veterinary Parasitology, 228, 126–129. 27692313 10.1016/j.vetpar.2016.09.001

[38] Jokelainen P, Tagel M, Mõtus K, Viltrop A, Lassen B. 2017 *Toxoplasma gondii* seroprevalence in dairy and beef cattle: large-scale epidemiological study in Estonia. Veterinary Parasitology, 236, 137–143. 28288757 10.1016/j.vetpar.2017.02.014

[39] Khames M, Yekkour F, Fernández-Rubio C, Aubert D, Nguewa P, Villena I. 2018 Serological survey of cattle toxoplasmosis in Medea, Algeria. Veterinary Parasitology Regional Studies Reports, 12, 89–90. 10.1016/j.vprsr.2018.02.009 31014815

[40] Klun I, Djurković-Djaković O, Katić-Radivojević S, Nikolić A. 2006 Cross-sectional survey on *Toxoplasma gondii* infection in cattle, sheep and pigs in Serbia: seroprevalence and risk factors. Veterinary Parasitology, 135(2), 121–131. 16188388 10.1016/j.vetpar.2005.08.010

[41] Lopes AP, Dubey JP, Neto F, Rodrigues A, Martins T, Rodrigues M, Cardoso L. 2013 Seroprevalence of *Toxoplasma gondii* infection in cattle, sheep, goats and pigs from the North of Portugal for human consumption. Veterinary Parasitology, 193(1–3), 266–269. 23290614 10.1016/j.vetpar.2012.12.001

[42] Lumley T. 2004 Analysis of complex survey samples. Journal of Statistical Software, 9(1), 1–19.

[43] Lumley T. 2010 Complex surveys, in Wiley series in survey methodology, NJ, USA: John Wiley & Sons, Inc. p. 276.

[44] Lumley T, Scott AJ. 2015 AIC and BIC for modelling with complex survey data. Journal of Survey Statistics and Methodology, 3(1), 1–18.

[45] Matsuo K, Kamai R, Uetsu H, Goto H, Takashima Y, Nagamune K. 2014 Seroprevalence of *Toxoplasma gondii* infection in cattle, horses, pigs and chickens in Japan. Parasitology International, 63(4), 638–639. 24780140 10.1016/j.parint.2014.04.003

[46] Nöckler K, Serrano FJ, Boireau P, Kapel CM, Pozio E. 2005 Experimental studies in pigs on *Trichinella* detection in different diagnostic matrices. Veterinary Parasitology, 132(1–2), 85–90. 15985334 10.1016/j.vetpar.2005.05.033

[47] Olsen A, Berg R, Tagel M, Must K, Deksne G, Enemark HL, Alban L, Johansen MV, Nielsen HV, Sandberg M, Lundén A, Stensvold CR, Pires SM, Jokelainen P. 2019 Seroprevalence of *Toxoplasma gondii* in domestic pigs, sheep, cattle, wild boars, and moose in the Nordic-Baltic region: A systematic review and meta-analysis. Parasite Epidemiology Control, 5, e00100. 30906889 10.1016/j.parepi.2019.e00100 PMC6411595

[48] Opsteegh M, Schares G, Blaga R, van der Giessen J. 2016 Experimental studies on *Toxoplasma gondii* in the main livestock species (GP/EFSA/BIOHAZ/2013/01) Final report. p. 161 EFSA Supporting Publications, 2016:EN-995.

[49] Opsteegh M, Teunis P, Zuchner L, Koets A, Langelaar M, van der Giessen J. 2011 Low predictive value of seroprevalence of *Toxoplasma gondii* in cattle for detection of parasite DNA. International Journal for Parasitology, 41(3–4), 343–354. 21145321 10.1016/j.ijpara.2010.10.006

[50] Opsteegh M, Spano F, Aubert D, Balea A, Burrells A, Cherchi S, Cornelissen JBWJ, Dam-Deisz C, Guitian J, Györke A, Innes EA, Katzer F, Limon G, Possenti A, Pozio E, Schares G, Villena I, Wisselink HJ, van der Giessen JWB. 2019 The relationship between the presence of antibodies and direct detection of *Toxoplasma gondii* in slaughtered calves and cattle in four European countries. International Journal for Parasitology, 49(7), 515–522. 31108097 10.1016/j.ijpara.2019.01.005

[51] Pop A, Oprisan A, Pop A, Cerbu A, Stavarache M, Nitu R. 1989 Toxoplasmosis prevalence parasitologically evaluated in meat animals. Archives Roumaines de Pathologie Experimentale et Microbiologie, 48(4), 373–378. 2520675

[52] Rozette L, Dumètre A, Couquet CY, Dardé ML. 2005 Seroprevalence de la toxoplasmose chez des ovins et des bovins en Haute-Vienne. Epidemiologie et Santé Animale, 48, 97–99.

[53] Sibley LD, Khan A, Ajioka JW, Rosenthal BM. 2009 Genetic diversity of *Toxoplasma gondii* in animals and humans. Philosophical Transactions of the Royal Society B: Biological Sciences, 364(1530), 2749–2761. 10.1098/rstb.2009.0087 PMC2865090 19687043

[54] Smith JL. 1993 Documented outbreaks of toxoplasmosis: transmission of *Toxoplasma gondii* to humans. Journal of Food Protection, 56, 630–639. 31113044 10.4315/0362-028X-56.7.630

[55] Tan QD, Yang XY, Yin MY, Hu LY, Qin SY, Wang JL, Zhou DH, Zhu XQ. 2015 Seroprevalence and correlates of *Toxoplasma gondii* infection in dairy cattle in northwest China. Acta Parasitologica, 60(4), 618–621. 26408581 10.1515/ap-2015-0087

[56] Tenter AM. 2009 *Toxoplasma gondii* in animals used for human consumption. Memorias do Instituto Oswaldo Cruz, 104(2), 364–369. 19430665 10.1590/s0074-02762009000200033

[57] Tenter AM, Heckeroth AR, Weiss LM. 2000 *Toxoplasma gondii*: from animals to humans. International Journal for Parasitology, 30(12–13), 1217–1258. 11113252 10.1016/s0020-7519(00)00124-7 PMC3109627

[58] Tourdjman MTC, De Valk H, Goulet V, Le Strat Y. 2015 Toxoplasmose chez les femmes enceintes en France : évolution de la séroprévalence et des facteurs associés entre 1995 et 2010, à partir des Enquêtes nationales périnatales. Bulletin Épidémiologique Hebdomadaire, 15–16, 264–272.

[59] Villena I, Aubert D, Gomis P, Ferté H, Inglard JC, Denis-Bisiaux H, Dondon JM, Pisano E, Ortis N, Pinon JM. 2004 Evaluation of a strategy for *Toxoplasma gondii* oocyst detection in water. Applied Environmental Microbiology, 70, 4035–4039. 15240280 10.1128/AEM.70.7.4035-4039.2004 PMC444816

[60] Waldeland H. 1977 Toxoplasmosis in sheep. I. Long-term epidemiological studies in four breeding flocks. II. Influence of various factors on the antibody contents. III. Hematological, serological and parasitological studies. Acta Veterinaria Scandinavica, 18, 227–256. 560112 10.1186/BF03548451 PMC8377661

