# Supplementary material for: Toxoplasma gondii in beef consumed in France: regional variation in seroprevalence and parasite isolation
Source: Parasite. 2019 Dec 23;26:77. doi: 10.1051/parasite/2019076 (PMC6927255; doi:10.1051/parasite/2019076)
Supplement: Supplementary file 1 — Table. Number of collected/bioassayed samples of French origin, according to the terminal titer and the age of animals. [file parasite-26-77-olm.pdf]

Age (months)	Number of collected (bioassay) samples	**Number of collected (bioassay) samples with terminal titer of :**							
		0	*6*	*10*	*25*	*50*	*100*	*200*	*400*
< 8	574 (10)	535(3)	25(4)	6(1)	4(1)	3(0)	1(1)	0(0)	0(0)
8-12	33(3)	29(0)	2(1)	1(1)**	1(1)	0(0)	0(0)	0(0)	0(0)
13-24	602(44)	472(1)	88(13)	15(12)	22(15)	2(0)	2(2)	1(1)	0(0)
25-36	232(28)	178(1)	25(3)	16(12)	8(8)	3(3)	2(1)	0(0)	0(0)
37-48	207(29)	143(1)	40(6)	11(9)	5(5)	2(2)	3(3)	3(3)	0(0)
49-60	144(16)	107(0)	24(4)	6(5)	3(3)***	3(3)	0(0)	1(1)	0(0)
61-72	122(11)	98(0)	14(3)	5(3)	3(3)	0(0)	1(1)	1(1)	0(0)
73-84	88(11)	64(2)	18(4)	4(4)**	0(0)	0(0)	1(1)	1(0)	0(0)
85-96	110(21)	70(0)	21(6)	12(9)***	4(4)	1(1)	1(0)	0(0)	1(1)
97-108	65(10)	49(0)	5(1)	6(5)	3(3)	0(0)	1(0)	1(1)	0(0)
109-120	47(8)	32(1)	9(1)	4(4)	2(2)	0(0)	0(0)	0(0)	0(0)
121-132	40(7)	27(0)	7(1)	5(5)**	1(1)**	0(0)	0(0)	0(0)	0(0)
133-144	28(4)	19(0)	5(1)	3(2)	0(0)	1(1)	0(0)	0(0)	0(0)
145-156	12(3)	9(0)	3(3)	0(0)	0(0)	0(0)	0(0)	0(0)	0(0)
157-168	12(2)	8(0)	3(1)	1(1)	0(0)	0(0)	0(0)	0(0)	0(0)
169-180	7(0)	6(0)	1(0)	0(0)	0(0)	0(0)	0(0)	0(0)	0(0)
181-192	4(1)	3(0)	0(0)	1(1)	0(0)	0(0)	0(0)	0(0)	0(0)
193-204	4(0)	4(0)	0(0)	0(0)	0(0)	0(0)	0(0)	0(0)	0(0)
>205	6(1)	5(0)	1(1)	0(0)	0(0)	0(0)	0(0)	0(0)	0(0)
Adults without mentioned age	11(0)	11(0)	0(0)	0(0)	0(0)	0(0)	0(0)	0(0)	0(0)
TOTAL	2349*(209)								

* One animal without indication (adult/calf)

** All 3 mice died in 48h p.i.

*** Positive sample in bioassay

Table 1: Number of collected/bioassayed samples of French origin, according to the terminal titer and the age of animals.
